# Clinical Outcomes of High-Dose Aflibercept 8 mg in Polypoidal Choroidal Vasculopathy: A Systematic Review and Meta-Analysis

**DOI:** 10.7759/cureus.106769

**Published:** 2026-04-10

**Authors:** Abdullah Bousamri, Mohammad Al-shehri, Mohammad Mestarihi, Talal B Almutairi, Mateb Alaredhi, Bader Mahanna, Faisal Alharbi

**Affiliations:** 1 Ophthalmology, Jordan University of Science and Technology, Irbid, JOR; 2 Ophthalmology, Gulf Medical University, Ajman, ARE; 3 Ophthalmology, Farwaniya Hospital, Kuwait City, KWT

**Keywords:** aflibercept 8mg, anti-vegf therapy, best corrected visual acuity (bcva), meta-analysis, neovascular age-related macular degeneration (namd), polypoidal choroidal vasculopathy, polyp regression, systematic review

## Abstract

Polypoidal choroidal vasculopathy (PCV) is a distinct subtype of neovascular macular disease with variable responses to anti-vascular endothelial growth factor (anti-VEGF) therapy. This systematic review and meta-analysis evaluated the visual, anatomical, and lesion-regression outcomes of intravitreal aflibercept 8 mg, a formulation developed to extend dosing intervals while maintaining efficacy, in patients with PCV. PubMed, Embase, Scopus, Web of Science, and Google Scholar were searched from inception to March 12, 2026 (PROSPERO: CRD420261339679). Eligible studies enrolled adults with indocyanine green angiography (ICGA)-confirmed PCV treated with aflibercept 8 mg and reported at least one co-primary outcome: change in best-corrected visual acuity (BCVA), central retinal or subfield thickness (CRT/CST), or complete polyp regression. Risk of bias was assessed using the Cochrane Risk of Bias 2 (RoB 2), Risk Of Bias In Non-randomized Studies - of Interventions (ROBINS-I), and Joanna Briggs Institute (JBI) appraisal tools. Complete polyp regression was analyzed using a generalized linear mixed model with a logit link; continuous outcomes were pooled using restricted maximum likelihood estimation with the Hartung-Knapp-Sidik-Jonkman correction. Certainty of evidence was evaluated using the Grading of Recommendations Assessment, Development, and Evaluation (GRADE) framework. Five studies encompassing 362 eyes (245 treated with aflibercept 8 mg) were included. The pooled complete polyp regression rate was 57% (95% confidence interval (CI), 39%-73%; I^2^=55.9%; k=3). The pooled mean CRT/CST change was -157.8 µm (95% CI, -176.9 to -138.7 µm; I^2^=0%; k=2), though the estimate was predominantly driven by a single study contributing 95.5% of the inverse-variance weight. BCVA outcomes were not pooled due to metric incompatibility and clinical heterogeneity; however, individual study results were directionally favorable, with Early Treatment Diabetic Retinopathy Study (ETDRS) gains of +8.4 to +9.5 letters at 48 weeks in the largest contributing study. Extended dosing intervals were maintained in 80.5% (q12) and 86.5% (q16) of treated eyes at 48 weeks. One study reported intraocular inflammation in 10.8% of the overall neovascular age-related macular degeneration (AMD) cohort in that study, a safety signal not observed in the remaining studies. The certainty of evidence was low for CRT/CST change and very low for polyp regression and BCVA. Available evidence suggests that aflibercept 8 mg may produce clinically relevant polyp regression and substantial anatomical improvement in PCV, with a potential reduction in injection frequency. However, given the low to very low certainty of the current evidence base across all co-primary outcomes, definitive efficacy and safety conclusions cannot yet be drawn. Prospective, PCV-specific trials with standardized outcome reporting are needed.

## Introduction and background

Polypoidal choroidal vasculopathy (PCV) is a subtype of neovascular macular disease characterized by branching vascular networks with aneurysmal, polyp-like dilations, usually identified on indocyanine green angiography (ICGA) [1]. Although it is often grouped with neovascular age-related macular degeneration (nAMD), PCV behaves differently. The lesions look different, show distinct imaging features, vary across populations, and do not always respond to treatment in the same way [2]. In practice, treatment is not only aimed at stabilizing vision and reducing fluid, but also at achieving regression of the polypoidal lesions themselves, as persistent polyps are associated with recurrent hemorrhage and long-term visual decline [3]. It is particularly prevalent among East Asian populations, in whom PCV may account for up to 55% of all neovascular macular disease presentations, compared with 8%-15% in European cohorts [4].

Intravitreal anti-vascular endothelial growth factor (VEGF) therapy remains the backbone of PCV management [3]. Both clinical trials and real-world studies have shown that agents such as ranibizumab, aflibercept 2 mg, and brolucizumab can improve retinal structure and visual acuity [5,6]. Photodynamic therapy (PDT) with verteporfin has also been used in PCV, either as monotherapy or in combination with anti-VEGF agents, and has been shown to enhance polyp regression rates, particularly when used as adjunctive therapy [5,6]. Even so, not all patients respond fully. Some continue to have persistent or recurrent fluid, and complete polyp regression is not always achieved with anti-VEGF alone [6]. The need for frequent injections also adds a considerable treatment burden over time. These issues have pushed research toward therapies that can maintain outcomes while reducing how often patients need treatment [7].

Aflibercept 8 mg was developed with this goal in mind. As a higher-dose formulation, it is intended to extend dosing intervals without sacrificing efficacy [8]. In phase 3 trials for nAMD, it produced visual outcomes comparable to standard dosing, while allowing injection intervals of up to 12 or even 16 weeks [8]. The PULSAR (neovascular AMD) and PHOTON (diabetic macular oedema) phase 3 trials demonstrated non-inferiority of extended-interval aflibercept 8 mg to standard dosing, with approximately 80% of patients maintaining planned extended intervals at one year [8,9]. Given the role of VEGF in PCV, there is a clear rationale for using a higher dose to improve fluid control, reduce retinal thickness, and possibly increase rates of polyp regression, all while easing the treatment schedule [5].

So far, the evidence for aflibercept 8 mg in PCV is limited and somewhat mixed. Available studies range from post hoc trial analyses to retrospective cohorts and small observational series, often within broader nAMD populations that include PCV subgroups [8]. They report outcomes such as changes in best-corrected visual acuity (BCVA), retinal thickness, polyp regression, and fluid resolution. Still, most studies are small, follow-up periods are inconsistent, and outcomes are not always reported consistently. Direct comparisons with other anti-VEGF agents, such as aflibercept 2 mg, brolucizumab, and faricimab, are scarce and typically derive from single-cohort studies rather than combined analyses [4].

To our knowledge, this is the first systematic review and meta-analysis evaluating intravitreal aflibercept 8 mg in PCV. This study aims to synthesize the available evidence on visual, anatomical, and polyp regression outcomes associated with aflibercept 8 mg. Where quantitative synthesis is feasible, pooled estimates are reported for BCVA, central retinal or subfield thickness, and complete polyp regression. Secondary outcomes include dry macula or fluid-resolution rates, treatment durability, and ocular safety. When direct comparative data are available, outcomes are also examined relative to other anti-VEGF therapies in exploratory analyses.

## Review

Methods

Protocol and Registration

This systematic review and meta-analysis was prospectively registered on PROSPERO (CRD420261339679). No amendments were made to the protocol after registration. The study adhered to these two tools: Preferred Reporting Items for Systematic Reviews and Meta-Analyses (PRISMA) 2020 and Cochrane guidelines [10,11].

Eligibility Criteria

We included studies of adults with PCV confirmed by ICGA or another accepted imaging method. Patients had to be treated with intravitreal aflibercept 8 mg, and each study needed to report at least one outcome: changes in BCVA, central retinal or subfield thickness (CRT/CST), or complete polyp regression.

Eligible designs included randomized controlled trials (RCTs), cohort studies (prospective or retrospective), post hoc subgroup analyses with PCV-specific data, and case series with measurable results. Studies with mixed nAMD populations were only included if PCV data could be clearly separated. We excluded studies without usable PCV-specific outcomes, as well as reviews, editorials, conference abstracts without sufficient data, and duplicate cohorts that did not provide additional information.

Information Sources and Search Strategy

We searched PubMed/Medline, Embase, Scopus, and Web of Science from inception to March 12, 2026, with no language restrictions. The strategy combined controlled vocabulary (e.g., medical subject headings (MeSH) and Emtree) with free-text terms for PCV and aflibercept. We did not restrict by dose, and studies of aflibercept 8 mg were identified during screening. Google Scholar was searched by relevance, and the first 200 results were reviewed. Full search strategies for each database are provided in Appendix 1.

Study Selection

Retrieved records were imported into EndNote X9 (Clarivate, St. Helier, Jersey, UK) for deduplication and management. Two reviewers independently screened titles and abstracts, followed by full-text assessment of potentially eligible records. Disagreements were resolved through discussion with a third reviewer. Eligibility was determined at the outcome level; therefore, individual studies could contribute to some analyses but not others if PCV-specific data were not available for all outcomes.

Data Extraction

Extraction was conducted independently by two reviewers using a standardized, pilot-tested extraction form. The form captured five domains: (1) study characteristics (author, year, country, design); (2) population characteristics (total and PCV-specific sample size, diagnosis method, baseline BCVA and CRT/CST); (3) intervention characteristics (drug, dose, loading and maintenance regimen); (4) outcome data (BCVA change, CRT/CST change, polyp regression, dry macula rate, and safety events); and (5) outcome definitions and measurement (imaging modality for polyp assessment, definition of polyp regression, and reported precision terms including standard deviation (SD), standard error (SE), and confidence interval (CI)). Discrepancies were resolved by a third-reviewer adjudication.

For continuous outcomes, baseline and follow-up means with SDs, mean change from baseline, and available precision terms were recorded. The BCVA measurement metric (logMAR (logarithm of the minimum angle of resolution) or Early Treatment Diabetic Retinopathy Study (ETDRS) letters) and anatomical thickness label (CRT, CST, or central foveal thickness (CFT)) were recorded for each study and applied as stratifying variables in subsequent analyses. For binary outcomes, event counts and denominators were extracted alongside the imaging modality and definition used to confirm polyp regression. Dry macula was defined as the absence of intraretinal and subretinal fluid on optical coherence tomography; where studies used alternative definitions such as absence of macular fluid, this was noted and reported descriptively.

When SDs of change were not directly reported, they were derived using a prespecified hierarchy: extraction of reported SD or SE, back-calculation from 95% CI, or imputation from baseline and follow-up SDs using the Cochrane-recommended approach, assuming a pre-post correlation coefficient (r) of 0.5, with sensitivity analyses at r=0.3 and r=0.7 [11]. Study-specific variance imputation procedures are detailed in Appendix 2. For studies reporting least-squares (LS) mean changes from mixed models for repeated measures, the SE was derived from the reported 95% CI and entered directly as the study-level precision term without further transformation.

Risk of Bias Assessment

Risk of bias was assessed independently by two reviewers at the outcome level using design-specific tools, with disagreements resolved by a third reviewer. Randomized evidence was evaluated using the Cochrane Risk of Bias 2 (RoB 2) tool, assessing the effect of assignment to intervention across five domains: bias arising from the randomization process, deviations from intended interventions, missing outcome data, measurement of the outcome, and selection of the reported result [12]. Non-randomized comparative and switch-cohort studies were assessed using the Risk Of Bias In Non-randomized Studies - of Interventions (ROBINS-I) version 2 tool. For the switch-cohort design, a predefined target trial framework was applied to approximate a comparison between switching to aflibercept 8 mg and continuation of prior therapy. Assessments addressed bias due to confounding, selection of participants, deviations from intended interventions, missing data, measurement of outcomes, and selection of the reported result [13]. Uncontrolled interventional case series were evaluated using the Joanna Briggs Institute (JBI) Critical Appraisal Checklist for Case Series. Domain-level and overall judgments were assigned according to each tool's recommended algorithm and rating scale [14].

Data Synthesis and Statistical Analysis

All analyses were performed in R version 4.5.2 (R Foundation for Statistical Computing, Vienna, Austria) using the metafor and meta packages, and the robvis package was used for visualization of risk-of-bias assessments. A random-effects framework was applied throughout. Outcomes were classified as eligible for quantitative synthesis on a per-outcome basis if PCV-specific data were available or extractable, at least two independent data points were present, and outcome metrics were sufficiently comparable to justify pooling. Studies not meeting these criteria contributed to the qualitative synthesis only. The unit of analysis was the eye; all denominators and pooled estimates are reported at the eye level.

Complete polyp regression was analysed using a generalized linear mixed-effects model with a logit link function. A Freeman-Tukey double-arcsine transformation model was fitted as a prespecified sensitivity analysis. When multiple intervention arms from the same study were eligible, they were combined into a single study-level estimate to avoid double-counting; sensitivity analyses were conducted with arms entered separately. Continuous outcomes were analysed using restricted maximum likelihood (REML) estimation with the Hartung-Knapp-Sidik-Jonkman correction. BCVA outcomes were analyzed separately by measurement metric; logMAR and ETDRS-letter data were not pooled.

Between-study heterogeneity was quantified using I², τ², and Cochran’s Q. Prediction intervals were calculated for pooled estimates and interpreted in the context of the number of contributing studies and between-study variance. Heterogeneity estimates at k=2 are reported with caution due to known instability. Formal assessment of small-study effects was not performed due to the limited number of studies per outcome.

Comparator-specific findings were summarized separately for each anti-VEGF agent; no pooling across comparator drugs was performed. All comparative analyses were prespecified as exploratory.

Certainty Assessment

The certainty of evidence for co-primary outcomes was assessed using the Grading of Recommendations Assessment, Development, and Evaluation (GRADE) framework. Observational evidence was assigned a starting certainty of low; ratings were downgraded for serious or very serious concerns across five domains (risk of bias, inconsistency, indirectness, imprecision, and publication bias) and could be upgraded for large effect magnitude, dose-response gradients, or residual confounding favouring the null.

Results

Study Selection

**Figure 1:**
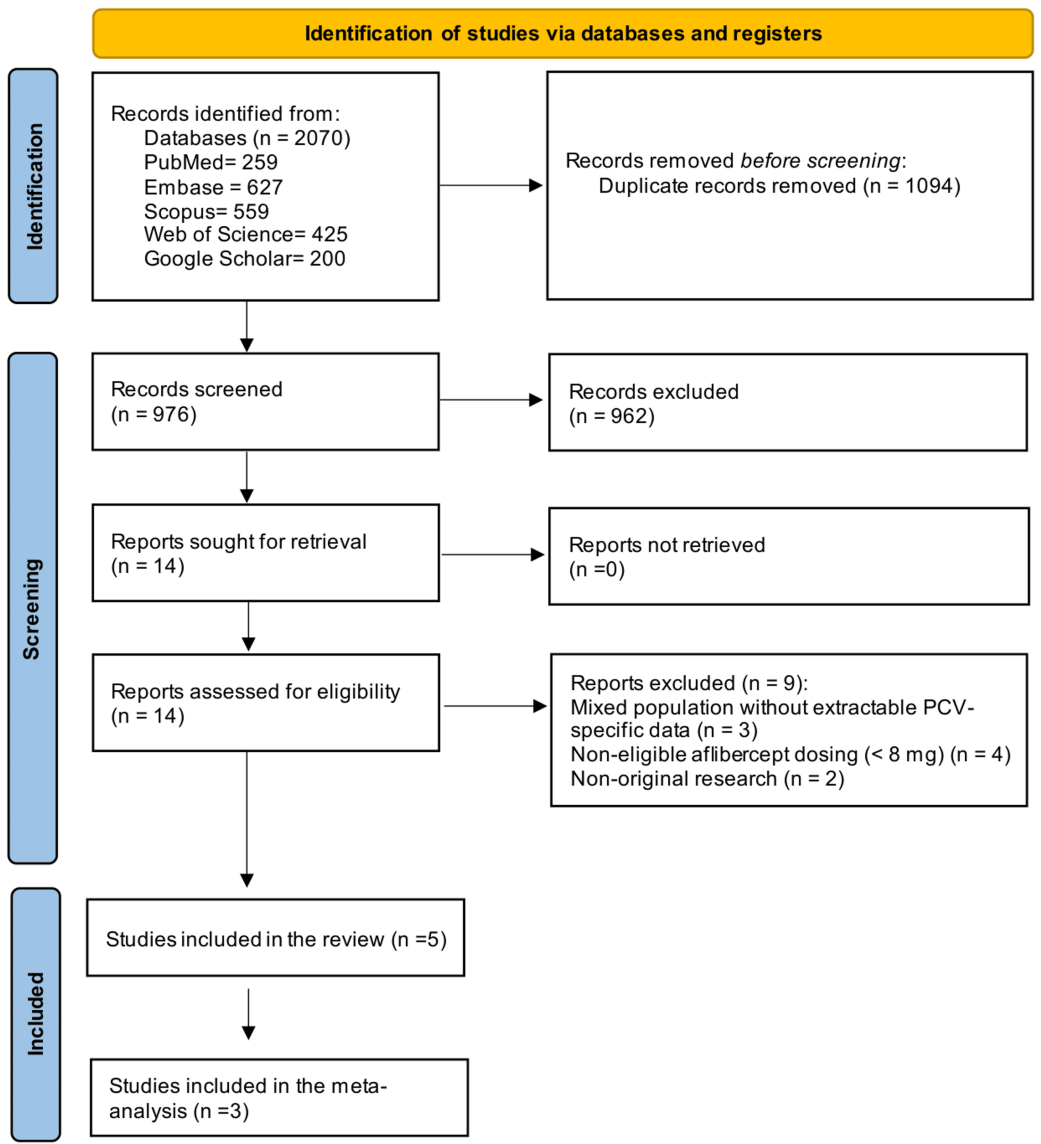
PRISMA 2020 flow diagram of the included studies. PRISMA: Preferred Reporting Items for Systemic Reviews and Meta-Analyses.

The database search identified 2,070 records, of which 976 remained after deduplication. Following title and abstract screening, 962 records were excluded and 14 full-text articles were assessed for eligibility. Nine were excluded at full-text review, most commonly due to the absence of PCV-specific extractable data, mixed nAMD populations without separable PCV subgroups, irrelevant intervention, and design. Five studies met all eligibility criteria and were included in the systematic review (n=5); three contributed to primary quantitative synthesis (meta-analysis), while two contributed to qualitative or exploratory analyses only. The study selection process is summarized in Figure 1.

Study Characteristics

Five studies encompassing 362 eyes (245 aflibercept 8 mg-treated) met the inclusion criteria (Table 1) [15-19]. One was a post hoc PCV subgroup analysis from the PULSAR phase 3 RCT (Lee et al. 2026 [15]; 48 weeks), and four were retrospective studies conducted in Japan and Singapore with a follow-up of 12 weeks to 12 months. Four studies enrolled treatment-naïve populations; one evaluated a switch cohort of previously treated eyes (Cheung et al. 2026 [16]), which did not contribute to pooled analyses owing to its design, lack of directly comparable PCV-specific data, and critical risk of bias under ROBINS-I. PCV was confirmed by ICGA in all the studies. Further baseline information is provided in Appendix 3.

**Table 1:** Study and Population Characteristics Abbreviations: ICGA, indocyanine green angiography; mg, milligrams; nAMD, neovascular age-related macular degeneration; PCV, polypoidal choroidal vasculopathy; q8/q12/q16, every 8/12/16 weeks; RCT, randomized controlled trial. ^a ^A total of 139 eyes were included across three treatment arms: aflibercept 8 mg every 12 weeks (q12; n = 44), aflibercept 8 mg every 16 weeks (q16; n=41), and aflibercept 2 mg every 8 weeks (q8; n=54).

Study	Country	Design	Population	Sample Size	Diagnosis	Treatment	Follow-up
Lee et al. (2026) [15]	Multinational	Post hoc subgroup of Phase 3 RCT (PULSAR)	Treatment-naïve PCV	139 eyes (139 PCV)^a^	ICGA	Aflibercept 8 mg q12 / q16 vs aflibercept 2 mg q8	48 weeks
Fukuda et al. (2025) [17]	Japan	Retrospective cohort	Treatment-naïve PCV	48 eyes (48 PCV)	ICGA	Aflibercept 8 mg vs brolucizumab 6 mg	3 months
Shiratori et al. (2026) [19]	Japan	Retrospective cohort	Treatment-naïve nAMD	62 eyes (22 PCV)	ICGA	Aflibercept 8 mg vs faricimab 6mg	16 weeks
Cheung et al. (2026) [16]	Singapore	Retrospective switch cohort	Previously treated nAMD	30 eyes (11 PCV)	ICGA	Switch to aflibercept 8 mg	Variable
Matsumoto et al. (2025) [18]	Japan	Retrospective case series	Treatment-naïve nAMD	83 eyes (21 PCV)	ICGA	Aflibercept 8 mg loading	12 weeks

Risk of Bias

**Figure 2:**
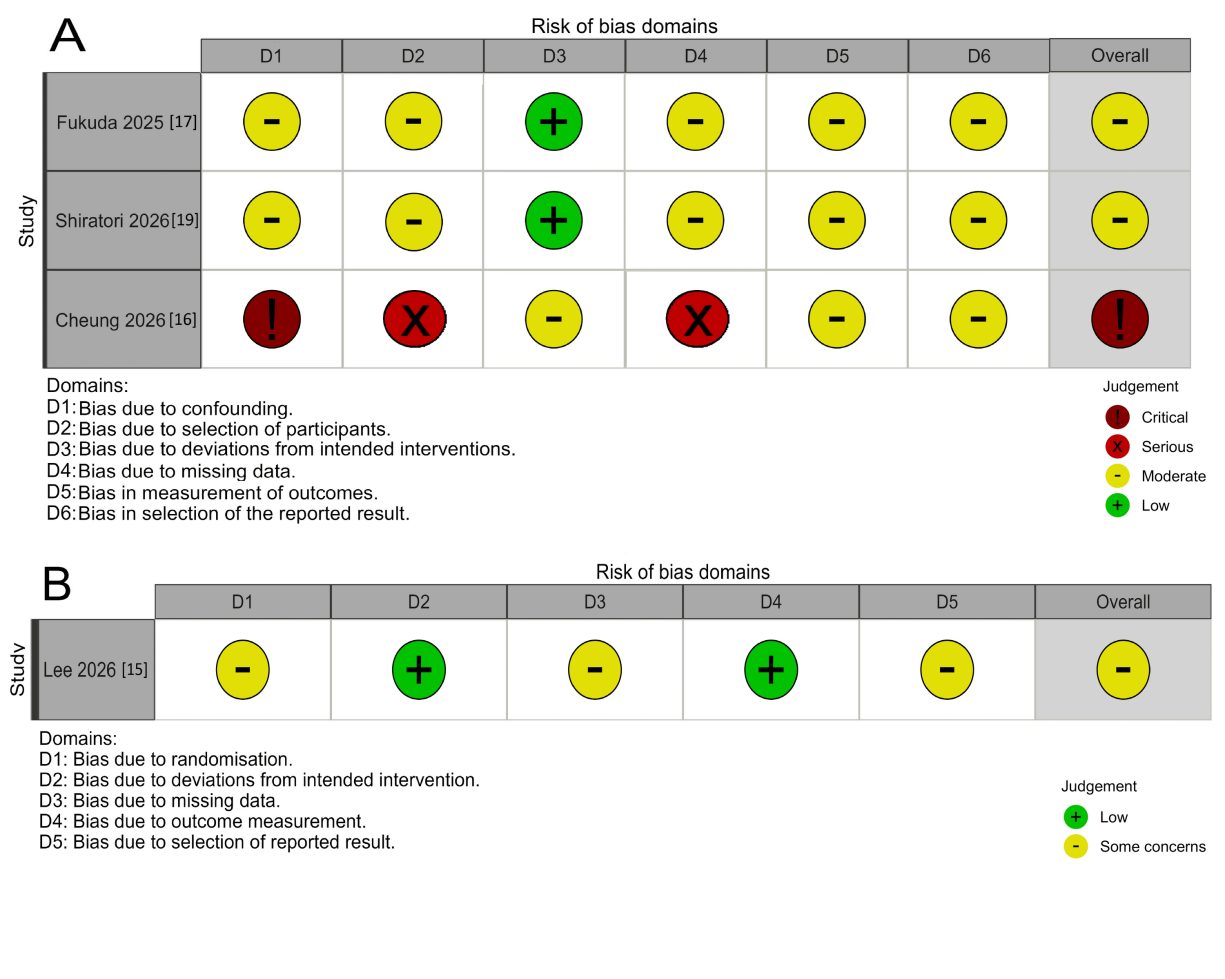
A: Risk of bias assessment using ROBINS-I for non-randomized studies. B: RoB 2 for the randomized subgroup analysis study. From Refs. [15-19]. ROBINS-I: Risk Of Bias In Non-randomized Studies - of Interventions.

**Figure 3:**
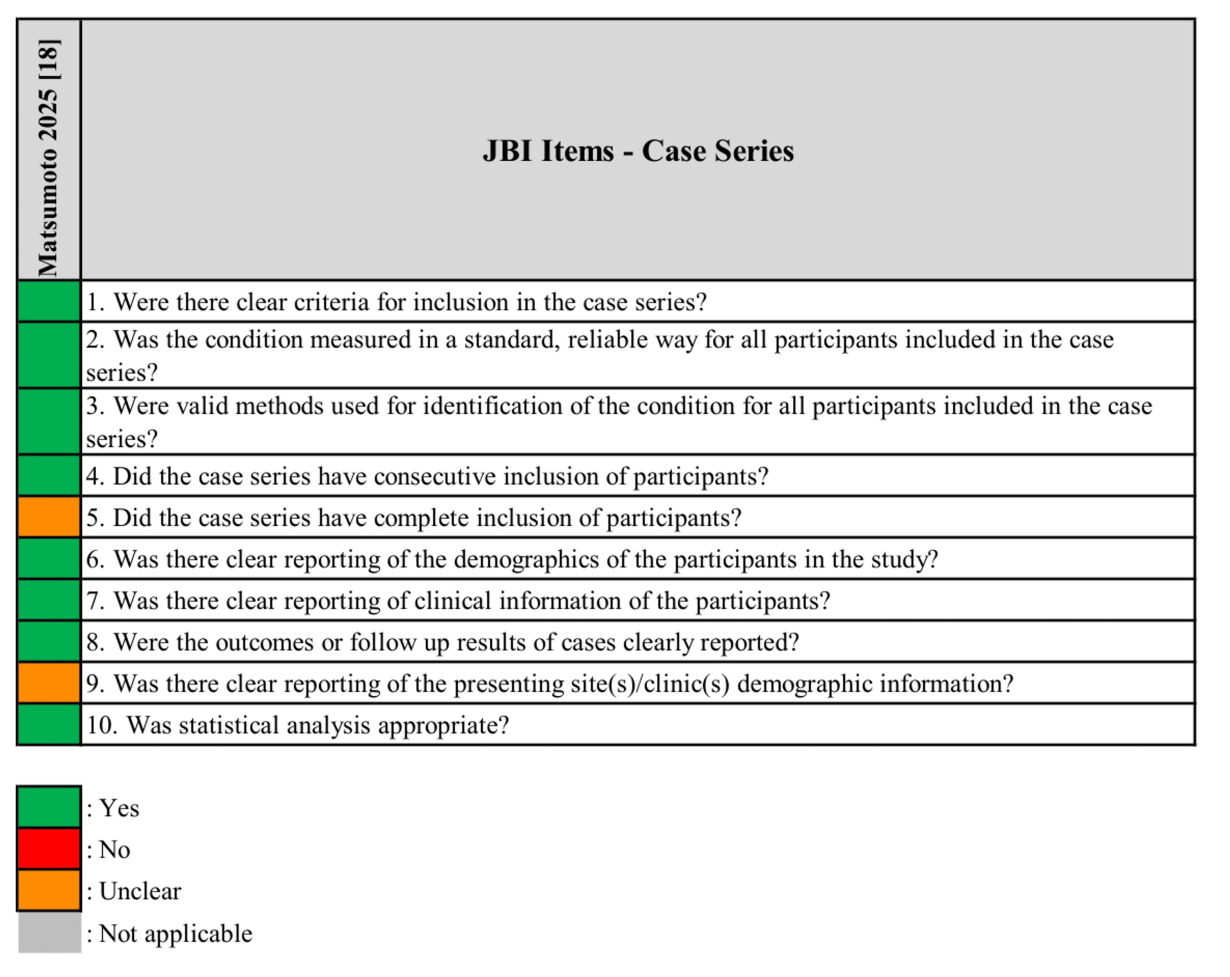
Risk of bias assessment using the Joanna Briggs Institute (JBI) Critical Appraisal Checklist for the Case Series [18]

Risk of bias varied across included studies (Figures 2, 3). The Lee et al. (2026) [15] study was judged to have concerns under RoB 2, identified across multiple domains. Fukuda et al. (2025) [17] and Shiratori et al. (2026) [19] were rated as having a moderate risk of bias under ROBINS-I. Cheung et al. (2026) [16] was judged to be at critical risk of bias, driven by uncontrolled confounding, serious selection bias, and incomplete outcome data. Matsumoto et al. (2025) [18], assessed using the JBI checklist, was of moderate-to-high quality with some unclear items related to case inclusion.

Primary Outcomes

**Figure 4:**
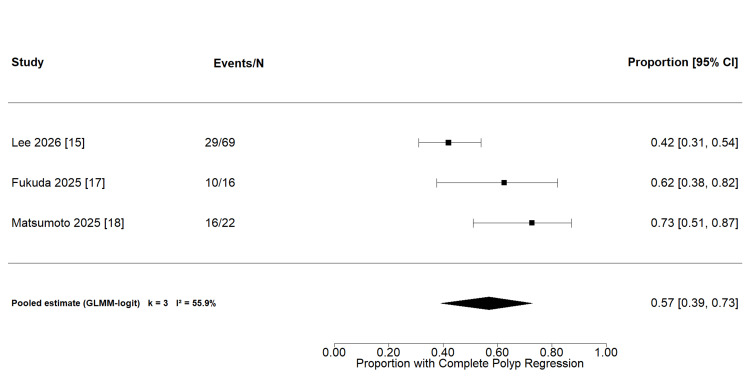
Forest plot of complete polyp regression rates following intravitreal aflibercept 8 mg in PCV (k=3; GLMM-logit model). Included studies [15,17,18]. GLMM: Generalized linear mixed model.

*Complete polyp regression*:* *Complete polyp regression, defined as the complete disappearance of polypoidal lesions on ICGA, was assessed in three studies encompassing 107 eyes, with follow-up durations ranging from 12 to 48 weeks. In Lee et al. [15], the regression analysis included 69 eyes with evaluable ICGA follow-up across the two aflibercept 8 mg arms, rather than the full 8 mg arm population. The pooled complete regression rate was 57% (95% CI 39-73%), with moderate between-study heterogeneity (I²=55.9%; Figure 4). A Freeman-Tukey double-arcsine model produced directionally consistent findings. Given that fewer than 10 studies were available, funnel plot-based assessment of small-study effects was not performed.

**Figure 5:**
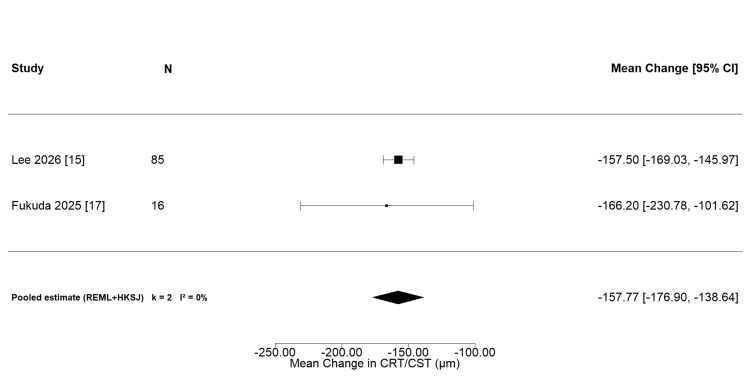
Forest plot of mean change in central retinal or subfield thickness following intravitreal aflibercept 8 mg in PCV (k=2). Included studies [15,17].

*Central retinal or subfield thickness change: *Two studies provided extractable PCV-specific data on central retinal or subfield thickness change following aflibercept 8 mg administration. Lee et al. [15] contributed a combined LS mean CST reduction of -157.5 µm derived from the PULSAR PCV subgroup, and Fukuda et al. [17] reported a mean CRT reduction of -166.2 µm at 12 weeks, with the SD of change imputed from baseline and follow-up SDs using a prespecified pre-post correlation of r=0.5. The pooled mean change was -157.8 µm (95% CI: -176.9 to -138.7 µm), with no between-study heterogeneity (I²=0%; Figure 5). Sensitivity analyses varying the assumed correlation coefficient (r=0.3 and r=0.7) did not materially alter this estimate. An exploratory three-study analysis incorporating Shiratori et al. [19], whose anatomical outcome was central foveal thickness, yielded a consistent pooled estimate of -158.8 µm (95% CI: -170.0 to -147.5 µm; I²=0%).

*Best-corrected visual acuity: *Visual acuity outcomes were analyzed separately by measurement metric. Lee et al. [15] was the only study reporting BCVA change in ETDRS-letter format; the LS mean gain was +9.5 letters (95% CI: 5.7-13.3) in the 8q12 arm and +8.4 letters (95% CI: 5.9-10.8) in the 8q16 arm at 48 weeks. As only one study contributed ETDRS-based data, no pooled estimate was computed for this metric.

For logMAR-based BCVA, two independent studies provided extractable data: Fukuda et al. [17] (pure PCV, treatment-naïve, 12 weeks, n=16) and Shiratori et al. [19] (PCV subgroup within a mixed nAMD cohort, 16 weeks, n=11). Fukuda et al. [17] reported a mean improvement of -0.10 logMAR, whereas Shiratori 2026 [19] reported a mean improvement of −0.30 logMAR. Although two datasets were available, quantitative synthesis was not performed because of substantial clinical heterogeneity, including differences in population composition, baseline BCVA, and follow-up duration. In addition, pooling at k=2 yielded unstable heterogeneity estimates and a non-informative Hartung-Knapp-Sidik-Jonkman confidence interval. BCVA findings were therefore summarized narratively.

Secondary Outcomes

*Dry macula and fluid resolution*:* *Formal meta-analysis was not feasible for this outcome. Fukuda et al. [17] provided a PCV-specific dry macula rate isolatable to the aflibercept 8 mg arm, with 93.8% (15/16) of treated eyes achieving a dry macula at three months. Lee et al. [15] reported PCV-specific fluid absence in 67% of aflibercept 8 mg-treated eyes by week 16, with proportions remaining in this range through week 48. The remaining studies reported dry macula or fluid resolution only for the overall nAMD cohort without separable PCV subgroup data and were therefore not eligible for PCV-specific synthesis (Table 2).

**Table 2:** Dry Macula or Fluid-Free Outcomes by Study Abbreviations: nAMD, neovascular age-related macular degeneration; N/A, not applicable; NR, not reported; PCV, polypoidal choroidal vasculopathy. ^a^Only study reporting a PCV-specific dry macula rate suitable for descriptive analysis. ^b^Denominator (70 eyes) reflects the subgroup with evaluable week-12 anatomical outcomes. ^c^Outcome defined as the absence of macular fluid rather than explicitly reported as dry macula.

Study	Population	Dry Macula / Fluid-Free Rate	Timepoint	PCV-Specific Data
Fukuda et al. (2025)ᵃ [17]	PCV-specific (n=16)	15/16 (93.8%)	3 months	Yes
Shiratori et al. (2026) [19]	Overall cohort, aflibercept arm (n=31)	25/31 (80.6%)	16 weeks	No, not isolated to the PCV subgroup
Matsumoto et al. (2025)^b^ [18]	Overall nAMD cohort (n=70)	58/70 (82.9%)	12 weeks	No, overall cohort
Lee et al. (2026)^c ^[15]	PULSAR PCV subgroup (n=139)	57/85 (67.1%) fluid-free (aflibercept 8 mg arms)	16–48 weeks	Yes
Cheung et al. (2026) [16]	Switch cohort (n=30, variable follow-up)	NR	Variable	N/A

Treatment Burden and Durability

Treatment burden data were most informatively reported in Lee et al. [15], where mean injection counts over 48 weeks were 6.1 (SD 0.4) and 5.1 (SD 0.5) in the 8q12 and 8q16 arms, respectively, compared with 7.0 (SD 0.2) in the aflibercept 2 mg 2q8 arm. Extended dosing intervals were maintained in 80.5% (33/41) and 86.5% (32/37) of eyes at 48 weeks [15]. In the Cheung et al. [16] switch cohort, judged to be at critical risk of bias, the median treatment interval increased from eight to nine weeks to 10 weeks after conversion, with 60% of patients achieving ≥12-week intervals.

Exploratory Comparator-Specific Analyses

Direct comparisons were available from three studies; per protocol, each comparator was analysed separately.

Aflibercept 8 mg versus brolucizumab 6 mg (Fukuda et al. [17]; n=16 vs 32; three months): Both agents produced significant CRT reductions. BCVA improved significantly from baseline within the aflibercept arm (paired test, p=0.002), but not brolucizumab (p=0.08). Complete polyp regression rates were 62.5% versus 75.0% (p=0.57), and dry macula rates were identical at 93.8%. No intraocular inflammation was recorded in the aflibercept arm; two inflammatory or vascular events occurred with brolucizumab, including one serious arterial occlusion.

Aflibercept 8 mg versus faricimab 6 mg (Shiratori et al. [19]; PCV subgroup, n=11 vs 11; 16 weeks): Both agents produced substantial central foveal thickness reductions (p<0.001 vs baseline for both). Direct BCVA comparison is limited by baseline imbalance (0.44±0.27 vs 0.26±0.22 logMAR). Polyp regression was not reported for the PCV subgroup.

Aflibercept 8 mg versus aflibercept 2 mg (Lee et al. [15]; PULSAR PCV subgroup): Visual and anatomical outcomes were broadly equivalent across all three arms at 48 weeks. LS mean differences in BCVA change versus the 2q8 arm were +0.4 letters (95% CI: -4.4 to +5.2) for 8q12 and −0.7 letters (95% CI: -4.6 to +3.2) for 8q16. LS mean differences in CST change were +2 µm (95% CI: -19 to +23) and +9 µm (95% CI: -21 to +39), respectively. Complete polyp regression rates were 37% (8q12), 47% (8q16), and 38% (2q8).

Safety

Ocular safety data were available across all five included studies; formal pooling was not performed given small sample sizes, heterogeneous adverse event definitions, and short follow-up durations (Table 3). No cases of occlusive retinal vasculitis or endophthalmitis were recorded in the aflibercept 8 mg arms. A single case of mild posterior uveitis was reported in the aflibercept 8 mg q12 arm of Lee et al. (1/44, 2.3%) [15]. One retinal pigment epithelium (RPE) tear occurred in the aflibercept arm of Shiratori et al.; the patient was excluded from the original study's final efficacy analysis by the investigators [19]. The most notable safety signal was reported by Matsumoto et al. [18], where nine of 83 eyes (10.8%; 95% CI: 5.1-19.6%) in the overall nAMD cohort developed retinal vasculitis or non-infectious intraocular inflammation during the aflibercept 8 mg loading phase; PCV-specific rates were not separately reported. No other study recorded intraocular inflammatory events in the aflibercept 8 mg arms.

**Table 3:** Adverse Events Across Eligible Studies Abbreviations: Afl, aflibercept; AE, adverse event; IOI, intraocular inflammation; IOP, intraocular pressure; NR, not reported; RPE, retinal pigment epithelium. ^a^The author excluded this case from the final efficacy analysis (n=31). ^b^Reported for the overall nAMD cohort rather than the isolated PCV subgroup.

Study	Treatment Arm	Follow-up	Adverse Event	Events (n/N, %)	Serious AE
Lee et al. [15]	Afl 8 mg (q12)	48 weeks	Chorioretinitis / posterior uveitis	1/44 (2.3%)	No
	Afl 8 mg (q12)	48 weeks	Macular hemorrhage	1/44 (2.3%)	Yes
	Afl 8 mg (q12)	48 weeks	Foveal hemorrhage	2/44 (4.5%)	No
	Afl 8 mg (q16)	48 weeks	Foveal hemorrhage	1/41 (2.4%)	No
	Afl 2 mg (q8)	48 weeks	Subretinal hemorrhage	1/54 (1.9%)	No
	All arms	48 weeks	Occlusive retinal vasculitis/endophthalmitis	0/139 (0%)	-
Fukuda et al. [17]	Afl 8 mg	3 months	Intraocular inflammation	0/16 (0%)	No
	Afl 8 mg	3 months	Systemic adverse events	0/16 (0%)	No
	Brolucizumab 6 mg	3 months	Anterior chamber inflammation	1/32 (3.1%)	NR
	Brolucizumab 6 mg	3 months	Arterial occlusion	1/32 (3.1%)	Yes
Shiratori et al. [19]	Afl 8 mg	16 weeks	RPE tear^a^	1/32 (3.1%)	NR
Cheung et al. [16]	Afl 8 mg	Variable	Endophthalmitis / uveitis / IOP elevation	0/30 (0%)	No
Matsumoto et al. [18]	Afl 8 mg	12 weeks	Retinal vasculitis / non-infectious IOI^b^	9/83 (10.8%)	No

Certainty of Evidence

The certainty of evidence was low for CRT/CST change and very low for complete polyp regression and BCVA change (Table 4). Polyp regression was downgraded for imprecision; BCVA was downgraded for both inconsistency and imprecision. Publication bias was not formally assessable for any outcome. Baseline demographic and clinical characteristics across study arms are provided in Table 5.

**Table 4:** GRADE Summary of Findings Abbreviations: BCVA, best-corrected visual acuity; CI, confidence interval; CRT, central retinal thickness; CST, central subfield thickness; ETDRS, Early Treatment Diabetic Retinopathy Study; PI, prediction interval; RCT, randomized controlled trial; wk, weeks. ^a^Lee et al. rated "some concerns" (RoB 2); Fukuda 2025 rated moderate (ROBINS-I); Matsumoto et al. rated moderate-to-high (JBI). Limitations partially captured by LOW starting certainty. Not downgraded, though borderline. ^b^I²=55.9%; estimates directionally consistent. Not downgraded. ^c^Downgraded one level for imprecision (wide CI: 39-73%; prediction interval 0.1-100%). ^d^Not assessed (fewer than 10 studies). ^e^Lee et al. rated "some concerns" (RoB 2); flagged domains unrelated to anatomical measurement. Not downgraded. ^f^I²=0%, not informative at k=2. Not downgraded. ^g^Not downgraded; estimate dominated by one study but consistent across studies. ^h^Different BCVA metrics (ETDRS vs logMAR); pooling not performed. ^i^Downgraded one level for inconsistency (heterogeneity and baseline imbalance). ^j^Downgraded one level for imprecision (small sample size, no pooled estimate).

Outcome	No. of studies (eyes)	Study design	Risk of bias	Inconsistency	Indirectness	Imprecision	Publication bias	Summary of findings	Certainty
Complete polyp regression [15,17,18]	3 (107)	1 post hoc RCT; 1 cohort; 1 case series	Not serious^a^	Not serious^b^	Not serious	Serious^c^	Not assessed^d^	Pooled 57% (95% CI 39-73%); I²=55.9%; PI 0.1-100% (uninformative)	⊕⊖⊖⊖ Very low
CRT/CST change [15,17]	2 (101)	1 post hoc RCT; 1 cohort	Not serious^e^	Not serious^f^	Not serious	Not serious^g^	Not assessed^d^	Pooled -157.8 µm (95% CI -176.9 to -138.7); I²=0%; single study contributes 95.5% of weight	⊕⊕⊖⊖ Low
BCVA change [15,17,19]	3^h^ (112)	1 post hoc RCT; 2 cohorts	Not serious	Serious^i^	Not serious	Serious^j^	Not assessed^d^	Not pooled. Lee: +9.5/+8.4 ETDRS letters (48 wk); Fukuda: -0.10 logMAR (12 wk); Shiratori: -0.30 logMAR (16 wk)	⊕⊖⊖⊖ Very low

Sensitivity Analyses

A prespecified sensitivity analysis for complete polyp regression using the Freeman-Tukey double-arcsine transformation produced directionally consistent findings. The back-transformed pooled proportion was 0.573 (95% CI: 0.375-0.761), with moderate-to-substantial between-study heterogeneity (I²=72.0%; τ²=0.0197). A prediction interval was not calculated owing to the small number of contributing studies (k=3). Leave-one-out analysis yielded estimates ranging from 0.491 (omitting Matsumoto et al.) to 0.685 (omitting Lee et al.), indicating no single study disproportionately influenced the direction of effect. The higher I² relative to the primary generalized linear mixed model (GLMM; 72.0% versus 55.9%) reflects known differences in variance estimation between transformation-based and likelihood-based approaches in small proportion meta-analyses [15,17,18].

An exploratory anatomical thickness analysis incorporating Shiratori et al. (k=3, REML) yielded a pooled mean change of -158.8 µm (95% CI: -170.0 to -147.5 µm; 95% prediction interval -231.7 to -85.8 µm), with negligible between-study heterogeneity (I²=0%; Q=1.968, df=2, p=0.374). Study-level contributions were: Lee et al. (-157.5 µm, SE 5.885, 95.5% weight), Fukuda et al. (-166.2 µm, SE 32.952, 3.0% weight), and Shiratori et al. (-223.0 µm, SE 46.955, 1.5% weight). The negligible difference from the primary k=2 estimate (-157.8 µm) reflects Shiratori's minimal contribution under inverse-variance weighting [15,17,19].

Discussion

This systematic review provides the first quantitative synthesis of clinical outcomes associated with intravitreal aflibercept 8 mg in polypoidal choroidal vasculopathy, consolidating evidence from five studies encompassing 245 treated eyes. Three principal findings emerge: complete polyp regression was achieved in an estimated 57% of treated eyes; central retinal or subfield thickness was reduced by a mean of approximately 158 µm; and visual acuity improvements were recorded across all contributing studies, with ETDRS letter gains of +8.4 to +9.5 in the PULSAR PCV subgroup at 48 weeks [15]. Aflibercept 8 mg additionally demonstrated a durability advantage over standard-dose aflibercept 2 mg, with a reduction of one to two injections per year and extended dosing maintained in more than 80% of treated PCV eyes [8,15]. These findings establish clinically meaningful activity for aflibercept 8 mg in PCV, though the evidence base remains nascent and insufficient to support definitive comparative efficacy conclusions.

Polyp Regression

The pooled complete polyp regression rate of 57% (95% CI: 39-73%) is broadly consistent with rates reported for intravitreal anti-VEGF monotherapy in PCV. The PLANET study is the largest randomized trial of aflibercept 2 mg in PCV, reporting complete polyp regression in 39% of eyes at week 52 in the monotherapy arm, increasing to 45% with rescue photodynamic therapy [6]. The EVEREST II trial similarly reported complete polyp regression in 34.7% of eyes receiving ranibizumab monotherapy at 12 months, rising to 69.3% with adjunctive photodynamic therapy [5].

In this context, the pooled estimate observed here appears numerically higher than rates reported for standard-dose anti-VEGF monotherapy. However, this difference should be interpreted cautiously, given cross-study heterogeneity in study design, follow-up duration, and outcome definitions. Direct comparative evidence from Lee et al. (PULSAR PCV subgroup) showed broadly similar regression rates between aflibercept 8 mg (37%-47%) and aflibercept 2 mg (38%), suggesting that dose escalation does not consistently translate into greater lesion-level regression within the timeframes studied [15]. This observation is consistent with the hypothesis that sustained VEGF suppression duration, rather than peak intravitreal drug concentration alone, may be the primary determinant of polypoidal lesion regression [8,20].

Between-study heterogeneity in regression rates was moderate (I²=55.9%), likely reflecting differences in baseline disease severity, polyp morphology, lesion activity, ICGA acquisition protocols, and definitions of complete versus partial regression [4]. Standardization of polyp regression endpoints, including consensus definitions, confirmatory imaging intervals, and grading criteria, remains a methodological priority in PCV research [3] and is reflected in the heterogeneous reporting observed across included studies. Follow-up duration also differed across contributing studies (12-48 weeks), and polyp regression is a time-dependent endpoint; the pooled estimate should therefore be interpreted as a weighted average across timepoints rather than a uniform treatment effect at a single timepoint.

A*natomical Outcomes and Pharmacodynamic Rationale*

The pooled mean reduction in central retinal or subfield thickness of -157.8 µm (95% CI: -176.9 to -138.7 µm) represents a large and clinically meaningful anatomical response, consistent with the proposed mechanism of prolonged VEGF suppression with high-dose aflibercept. In the broader nAMD population of the PULSAR trial, mean CST reductions of approximately 130-160 µm were reported at 48 weeks [8], suggesting that the magnitude of anatomical response in PCV is consistent with that in other nAMD subtypes. Unlike ranibizumab, which binds VEGF-A selectively, aflibercept functions as a fusion-protein decoy receptor targeting VEGF-A, VEGF-B, and placental growth factor pathways collectively implicated in the pathogenesis of type 1 choroidal neovascularization and the exudative activity characteristic of PCV [20,21]. The dry macula rate of 93.8% observed in Fukuda et al. [17] at three months is consistent with the pharmacodynamic rationale that higher intravitreal concentrations prolong the duration of VEGF suppression and defer fluid recurrence, a premise that underlies the interval-extension strategy of the aflibercept 8 mg programme [8]. However, the pooled anatomical estimate was predominantly driven by a single study (Lee et al., contributing 95.5% of the inverse-variance weight), and the result should be considered confirmatory of the PULSAR PCV subgroup finding rather than an independently derived synthesis.

Visual Acuity Outcomes

Visual acuity improvements were directionally consistent across all contributing studies. In the Lee et al. [15] PULSAR PCV subgroup, LS mean gains of +9.5 and +8.4 ETDRS letters were observed at 48 weeks for the 8q12 and 8q16 arms, respectively, with LS mean differences versus the aflibercept 2 mg 2q8 arm of only +0.4 and −0.7 letters, indicating that the higher dose did not produce meaningfully greater visual gains, consistent with the primary PULSAR non-inferiority finding [8]. In retrospective cohorts, logMAR improvements ranged from -0.10 (Fukuda 2025 [17], 12 weeks) to -0.30 (Shiratori et al. [19], 16 weeks); the larger improvement in Shiratori et al. is plausibly attributable to substantially worse mean baseline BCVA (0.44 logMAR), reflecting greater room for recovery rather than a superior treatment effect. Although two studies reported logMAR-based outcomes, quantitative synthesis was not performed because outcome metrics were not sufficiently comparable to justify pooling, as prespecified in the analytical framework, given differences in population composition, baseline BCVA, and follow-up duration, compounded by instability of random-effects estimation at k=2. The inability to pool BCVA outcomes across logMAR and ETDRS scales further reflects a longstanding methodological limitation in retinal clinical research that impairs cross-study evidence synthesis and underscores the need for harmonized functional outcome reporting in future PCV trials [3].

Treatment Durability and Injection Burden

Real-world evidence consistently demonstrates that injection burden is a major contributor to undertreatment and suboptimal visual outcomes in neovascular AMD management [7]. In the PULSAR PCV subgroup, mean annual injection counts of 5.1 and 6.1 for the 8q16 and 8q12 arms, respectively, represented a reduction of approximately one to two injections per year compared with the aflibercept 2 mg 2q8 arm (7.0 injections). Extended dosing intervals were maintained in 80.5% and 86.5% of PCV eyes on the 8q12 and 8q16 regimens at 48 weeks, respectively, indicating robust durability in this subtype [15]. In the switch cohort of Cheung et al., 60% of patients achieved intervals of ≥12 weeks following conversion from prior anti-VEGF therapy, providing preliminary descriptive evidence that interval extension may be achievable in pretreated populations [16]. However, this finding derives from a study at critical risk of bias and should be interpreted with caution. These findings suggest that the durability advantages demonstrated in the broader nAMD populations of the PULSAR and PHOTON programmes translate meaningfully to PCV, though real-world evidence with follow-up beyond 12 months is needed to confirm that interval extension is sustainable and does not compromise polyp regression durability over time.

Safety Profile and the Intraocular Inflammation Signal

The safety profile of aflibercept 8 mg was broadly reassuring across the four studies providing arm-specific data, with no cases of occlusive retinal vasculitis or endophthalmitis, events that have been a recognized safety concern with brolucizumab 6 mg [22], and only isolated reports of mild posterior uveitis and a single RPE tear. The principal safety concern identified in this review is the intraocular inflammation rate of 10.8% (9/83 eyes) reported by Matsumoto et al. [18] in the overall nAMD cohort following a three-injection loading protocol. This substantially exceeds the inflammation rates below 1% reported in the PULSAR and PHOTON pivotal trials and warrants careful contextualization [8,9]. The retrospective, single-centre design of Matsumoto et al., combined with the absence of PCV-specific event rates, limits direct attribution, and ascertainment biases cannot be excluded. Nevertheless, the biological plausibility of dose-related local inflammation with higher intravitreal protein concentrations cannot be dismissed [23], and the higher 8 mg dose achieves greater intravitreal molar concentration, providing the pharmacological basis for prolonged VEGF suppression and extended dosing intervals [8]. Prospective, systematically graded collection of ocular inflammatory events remains a priority for all future aflibercept 8 mg studies in PCV.

Limitations

This review has several important limitations. The total evidence base comprises only five studies and 245 aflibercept 8 mg-treated PCV eyes, with predominantly retrospective designs and follow-up periods of 12-16 weeks in most cohorts. The most robust data derive from a single post hoc randomized subgroup analysis rather than a prospective PCV-specific trial. Outcome definition heterogeneity was pervasive across BCVA scales, anatomical thickness labels, and polyp regression criteria. All analyses were conducted at the eye level; at least one study included bilateral cases (Matsumoto et al. [18]: 83 eyes from 80 patients), introducing potential non-independence that was not statistically adjusted for. Standard deviations of change required imputation in at least one study. Formal publication bias assessment was precluded by insufficient study numbers. The absence of follow-up data beyond 12 months means that polyp recurrence rates, long-term visual trajectories, and the sustained durability of extended dosing intervals in PCV remain unknown. Additionally, one included study contributing durability outcomes employed a switch-cohort design without a concurrent comparator and was judged to be at critical risk of bias, substantially limiting the interpretability of durability-related findings.

Future directions

Prospective, adequately powered trials designed specifically for PCV, with ICGA-confirmed polyp regression as a co-primary endpoint, follow-up of at least 96 weeks, and harmonized outcome reporting standards are urgently needed to establish the comparative effectiveness of aflibercept 8 mg relative to standard-dose anti-VEGF agents and novel dual-pathway inhibitors such as faricimab [24]. Given the disproportionate burden of PCV among East Asian populations, regionally designed and powered trials that mandate PCV-specific subgroup analyses should be prioritized alongside global evidence generation [4]. Rigorous, prospective intraocular inflammation surveillance, using standardized grading criteria and sufficient follow-up to capture delayed-onset events, is specifically warranted to resolve the uncertainty introduced by the Matsumoto et al. signal.

## Conclusions

In this systematic review and meta-analysis, intravitreal aflibercept 8 mg was associated with improved visual and anatomic outcomes in polypoidal choroidal vasculopathy. Visual acuity improved across studies, central retinal or subfield thickness decreased, and the pooled polyp regression rate was 57%. The evidence is limited. Certainty was low to very low, and safety concerns, including a possible intraocular inflammation signal, remain. Current data do not show clear superiority over existing anti-VEGF therapies, so these findings should be interpreted cautiously. Larger prospective PCV-specific studies, with standardized outcomes and longer follow-up, are needed to better define efficacy, safety, and durability.

## Appendices

Appendix 1: Full search strings

Searched from inception to March 12, 2026.

*Pubmed: *259 results

("Polypoidal Choroidal Vasculopathy"[Mesh] OR "polypoidal choroidal vasculopath*"[tiab] OR "idiopathic polypoidal choroidal vasculopath*"[tiab] OR "polypoidal choroidal neovasculari*"[tiab] OR "polypoidal CNV*"[tiab] OR "PCV"[tiab] OR "IPCV"[tiab]) AND ("aflibercept"[Supplementary Concept] OR "aflibercept"[tiab] OR "ziv-aflibercept"[tiab] OR "eylea"[tiab] OR "zaltrap"[tiab] OR "VEGF-Trap"[tiab] OR "VEGF Trap-Eye"[tiab] OR "AVE005"[tiab] OR "AVE-005"[tiab] OR "AVE 005"[tiab] OR "AVE0005"[tiab] OR "AVE-0005"[tiab] OR "AVE 0005"[tiab])

*Embase*:* *627 results

('polypoidal choroidal vasculopathy'/exp OR 'polypoidal choroidal vasculopath*':ti,ab OR 'idiopathic polypoidal choroidal vasculopath*':ti,ab OR 'polypoidal choroidal neovasculari*':ti,ab OR 'polypoidal CNV*':ti,ab OR 'PCV':ti,ab OR 'IPCV':ti,ab) AND ('aflibercept'/exp OR 'aflibercept':ti,ab OR 'ziv-aflibercept':ti,ab OR 'eylea':ti,ab OR 'zaltrap':ti,ab OR 'VEGF-Trap':ti,ab OR 'VEGF Trap-Eye':ti,ab OR 'AVE005':ti,ab OR 'AVE-005':ti,ab OR 'AVE 005':ti,ab OR 'AVE0005':ti,ab OR 'AVE-0005':ti,ab OR 'AVE 0005':ti,ab)

*Scopus: *559 results

(TITLE-ABS-KEY("polypoidal choroidal vasculopath*") OR TITLE-ABS-KEY("idiopathic polypoidal choroidal vasculopath*") OR TITLE-ABS-KEY("polypoidal choroidal neovasculari*") OR TITLE-ABS-KEY("polypoidal CNV*") OR TITLE-ABS-KEY("PCV") OR TITLE-ABS-KEY("IPCV")) AND (TITLE-ABS-KEY("aflibercept") OR TITLE-ABS-KEY("ziv-aflibercept") OR TITLE-ABS-KEY("eylea") OR TITLE-ABS-KEY("zaltrap") OR TITLE-ABS-KEY("VEGF-Trap") OR TITLE-ABS-KEY("VEGF Trap-Eye") OR TITLE-ABS-KEY("AVE005") OR TITLE-ABS-KEY("AVE-005") OR TITLE-ABS-KEY("AVE 005") OR TITLE-ABS-KEY("AVE0005") OR TITLE-ABS-KEY("AVE-0005") OR TITLE-ABS-KEY("AVE 0005"))

*Web of Science (WoS)*:* *425 results

TS=("polypoidal choroidal vasculopath*" OR "idiopathic polypoidal choroidal vasculopath*" OR "polypoidal choroidal neovasculari*" OR "polypoidal CNV*" OR "PCV" OR "IPCV") AND TS=("aflibercept" OR "ziv-aflibercept" OR "eylea" OR "zaltrap" OR "VEGF-Trap" OR "VEGF Trap-Eye" OR "AVE005" OR "AVE-005" OR "AVE 005" OR "AVE0005" OR "AVE-0005" OR "AVE 0005")

*Google Scholar:* 200 results sorted by relevance

"polypoidal choroidal vasculopathy" AND aflibercept

Appendix 2: Study-specific variance imputation

The SD of change was imputed using the standard Cochrane formula, assuming a pre-post correlation coefficient of r=0.5, a commonly used assumption when within-subject correlations are not reported [11].

Imputation of SD for CRT/CST change

Fukuda et al. (2025) [17]

Baseline SD=152.2 µm Follow-up SD=76.0 µm n=16 Assumed correlation coefficient r=0.5

SDchange=√(SDbaseline² + SDfollow-up² − 2 × r × SDbaseline × SDfollow-up) SDchange=√(152.2²+76.0²−2×0.5×152.2×76.0) SDchange=√17373.64 SDchange=131.81 µm

Standard error: SE=SDchange / √n SE=131.81 / √16 SE=32.95 µm

Shiratori et al. [19]

Baseline SD=177 µm Follow-up SD=116 µm n=11 Assumed correlation coefficient r=0.5

SDchange=√(177²+116²−2×0.5×177×116) SDchange=√24253 SDchange=155.73 µm

Standard error: SE=SDchange/√n SE=155.73/√11 SE=46.96 µm

Imputation of SD for BCVA change

Fukuda et al. [17]

Baseline SD=0.27 logMAR Follow-up SD=0.25 logMAR n=16 Assumed correlation coefficient r=0.5

SDchange=√(0.27²+0.25²−2×0.5×0.27×0.25) SDchange=√0.0679 SDchange=0.261 logMAR

Standard error: SE=SDchange/√n SE=0.261/√16 SE=0.065

Shiratori et al. [19]

Baseline SD=0.27 logMAR Follow-up SD=0.15 logMAR n=11 Assumed correlation coefficient r=0.5

SDchange=√(0.27²+0.15²−2×0.5×0.27×0.15) SDchange=√0.0549 SDchange=0.234 logMAR

Standard error: SE=SDchange / √n SE=0.234 / √11 SE=0.071

Appendix 3: Additional baseline information

**Table 5:** Baseline Demographics and Clinical Characteristics Abbreviations: BCVA, best-corrected visual acuity; CFT, central foveal thickness; CRT, central retinal thickness; CST, central subfield thickness; nAMD, neovascular age-related macular degeneration; PCV, polypoidal choroidal vasculopathy. Values are presented as mean±SD unless otherwise noted. CFT = central foveal thickness (reported in place of CRT/CST by some studies).

Study	Evaluated Cohort	Treatment Arm	Age (years)	Male (%)	Baseline BCVA	Baseline CRT/CST (µm)
Lee et al. (2026) [15]	PCV only	Afl 8 mg q12 (n=44)	72.2 ± 8.1	50	56.3±13.3 letters	392 ± 129
		Afl 8 mg q16 (n=41)	73.2 ± 8.7	63	60.1±11.5 letters	377 ± 139
		Afl 2 mg q8 (n=54)	72.6 ± 8.2	69	57.6±15.5 letters	378 ± 163
Fukuda et al. (2025) [17]	PCV only	Afl 8 mg (n=16)	73.9±9.2	68.8	0.28±0.27 logMAR	358±152
		Brolucizumab 6 mg (n=32)	75.4±8.0	75	0.35±0.26 logMAR	382±157
Shiratori et al. (2026) [19]	Overall nAMD	Afl 8 mg (n=31)	76.9±7.3	61.3	0.44±0.25 logMAR	447±157 (CFT)
		Faricimab 6 mg (n=31)	77.8 ± 7.1	55.2	0.40±0.29 logMAR	402±169 (CFT)
Matsumoto et al. (2025) [18]	Overall nAMD	Afl 8 mg (n=70)	76.3 ± 8.1	62.7	0.33±0.45 logMAR	313±135 (CFT)
Cheung et al. (2026) [16]	Overall nAMD	Switch to Afl 8 mg (n=30)	71.3 ± 7.1	55.2	0.36±0.27 logMAR	329±94
